# Association between atherogenic index of plasma and new onset of type 2 diabetes among elderly in China: a longitudinal study

**DOI:** 10.3389/fendo.2025.1632400

**Published:** 2025-10-15

**Authors:** Xiang-yang He, Zhi-wei Lu, Yan-Fang Guo, Ren-cheng Zhao, Zheng Liu

**Affiliations:** ^1^ Department of Health Management, Shenzhen Baoan District Chronic Diseases Prevent and Cure Hospital, Shenzhen, Guangdong, China; ^2^ Department of Health Education, Guangdong Health Promotion and Education Center, Guangzhou, Guangdong, China

**Keywords:** atherogenic index of plasma (AIP), type 2 diabetes (T2D), longitudinal study, elderly, Baoan health longitudinal study (BaHLS)

## Abstract

**Background:**

Previous studies have reported an association between the atherogenic index of plasma AIP and CVD incidence. However, few studies have investigated its longitudinal predictive effect on T2D among elderly individuals.

**Methods:**

Data from the BaHLS of the Community-Based Elderly Population in Shenzhen, China, were utilized. AIP was calculated as log10 ^[TG/HDL-C]. The subjects were divided into four groups on the basis of the quartiles of AIP values at baseline. A multivariable Cox proportional hazards model and a GAM were employed to assess the longitudinal predictive effect of the AIP on the risk of new-onset T2D. The predictive efficacy of the AIP, TG, and HDL-C for new-onset T2D was analyzed and compared via receiver operating characteristic (ROC) curve analysis.

**Results:**

A total of 18,295 subjects were selected, of whom 1,300 developed T2D by 2022. After adjusting for confounding factors, we found that the AIP was positively associated with the incidence of T2D. When AIP was categorized into four groups based on quartiles, the risk of new-onset T2D in the 2nd to 4th groups increased by 26% (95% *CI*: 4%, 52%), 40% (95% *CI*: 17%, 68%), and 57% (95% *CI*: 31%, 87%) compared with that in the 1st group, respectively. The AUCs for AIP, TG, and HDL-C were 0.601 (95% *CI*: 0.586 - 0.617; *p* < 0.001), 0.589 (95% *CI*: 0.573 - 0.605; *p* < 0.001), and 0.567 (95% *CI*: 0.551 - 0.584, *p* < 0.001), respectively. A nonlinear association was observed between the AIP score and the risk of new-onset T2D. In patients with an AIP ≤ 0, an increase in the AIP was significantly associated with an increased risk of T2D. The relationship between the AIP score and the risk of new-onset T2D persisted across most subgroups.

**Conclusion:**

The study demonstrated that the AIP was positively associated with an increased incidence of T2D, suggesting that the AIP should serve as a valuable indicator for monitoring and preventing new-onset T2D.

## Introduction

T2D is a prevalent metabolic disorder characterized by a complex interplay of endocrine-metabolic factors driven by excessive energy intake (1). With rapid economic development, significant lifestyle changes, and a rapidly aging population, type 2 diabetes (T2D) has become one of the major public health problems worldwide, particularly in developing countries (2, 3). According to the Diabetes Map (10th edition) published by the IDF, the number of individuals with diabetes reached 537 million (prevalence was 10.5%) in 2021. Additionally, approximately 6.7 million deaths were attributed to diabetes or its complications in 2021, and the prevalence will increase to 12.2% in 2025 (4, 5). The prevalence of diabetes is approximately 11% among Chinese adults (6). Epidemiological studies indicate that the older age group with a higher incidence of developing age-related conditions is those aged 65 years and above (2, 7).

AIP is an innovative lipid marker proposed by Dobiásová and Frohlich in 2001 (8). It is calculated through a logarithmic transition of the ratio of TG to HDL-C with molar concentrations. Elevated TG levels can lead to insulin resistance (9, 10), while HDL-C plays crucial antioxidant and anti-inflammatory roles in regulating metabolic disorders, including diabetes (11). AIP has been found to be negatively associated with the particle size of lipoproteins and FER (8). AIP was initially used as a biomarker for plasma atherosclerosis (8). Research has indicated that subjects with dyslipidemia have a greater risk of developing T2D (12). The AIP combines TG and HDL-C levels, reflecting not only their ratio but also the size of lipoprotein particles. This makes it an innovative biomarker that comprehensively responds to abnormalities in lipid metabolism and is simple to measure (13).

AIP was initially built to predict the risk of CVD (13, 14). Recently, interest in the association between AIP and metabolic diseases has increased (15–18). Few studies have examined the relationship between AIP and T2D, with most being cross-sectional and involving small sample sizes (fewer than 400 participants) (18–20). Additionally, there is limited research on the longitudinal predictive effect of the AIP on T2D in the elderly population (21, 22). Therefore, this study utilized a large cohort of elderly individuals in Chinese communities to explore the longitudinal association between the AIP and the risk of new-onset T2D. The purpose of this study was to identify a simple, easily measurable biomarker for effectively predicting T2D.

## Methods

### Data sources and study population

This study utilized data from the BaHLS of community-dwelling older adults in Shenzhen, China. The BaHLS conducted a baseline survey in 2020 (Wave 0), enrolling individuals aged 65 years and older. Participants were recruited on a voluntary basis by local community health centers. Eligibility criteria included residing in the local area for more than six months, and signing informed consent. Trained medical personnel from community service centers conducted one-on-one structured interviews and health check-ups with all participants. Information regarding socio-demographic factors, lifestyle choices, health status, and biological indicators were collected. A total of 22,809 subjects were surveyed at baseline. Annual one-on-one structured interviews and health check-ups were conducted during the follow-up period. Two follow-up surveys (Wave 1 in 2021, and Wave 2 in 2022) have been completed. Subjects were required to provide blood samples and sign informed consent during each follow-up survey.

**Figure 1:**
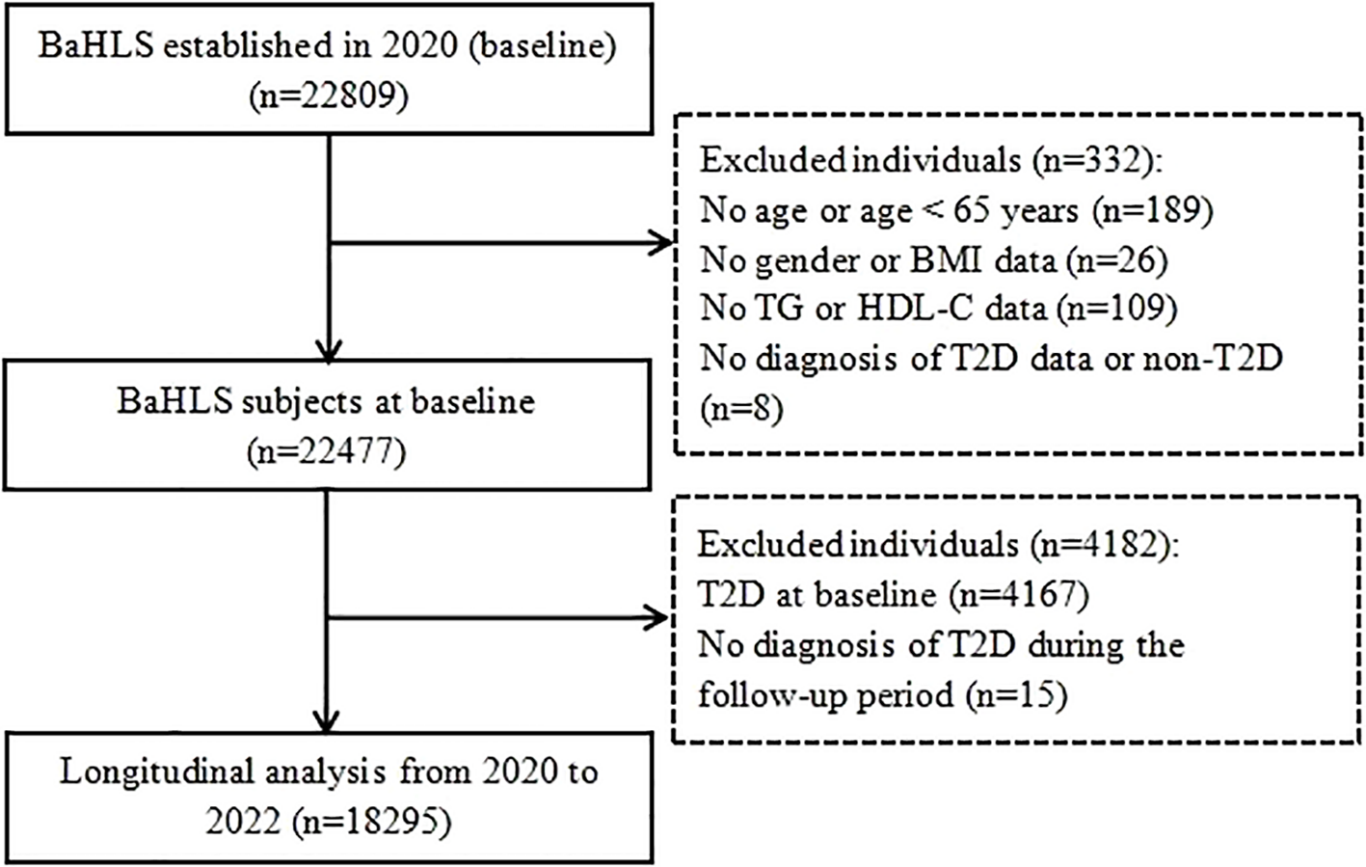
Flowchart of participants selection from BaHLS 2020-2022.

We utilized the BaHLS 2020 baseline data and the 2021-2022 follow-up data for this study. First, we excluded participants with missing age information or those under 65 years (n=189). Subsequently, we excluded individuals with missing data on gender, height, or weight (n=26). Additionally, we excluded subjects with incomplete exposure indicators for TG and/or HDL-C metrics (n=109). Participants with missing data on T2D diagnosis at baseline (n=8) were also excluded. We excluded subjects diagnosed with T2D at baseline (n=4,167) and those with missing information regarding T2D diagnosis during follow-up (n=15). Ultimately, 18,295 subjects with complete data were included in the analysis to examine the longitudinal association between AIP and the new onset of T2D. The detailed inclusion process of subjects is illustrated in **Figure 1**.

### Data collection

The BaHLS (from 2020 to 2022) was conducted by trained medical personnel from local community health centers. The primary sources of variables included standardized questionnaires, physical measurements and blood samples. The standardized questionnaire included demographic information (e.g., age, sex, education level), lifestyle behaviors (e.g., smoking, drinking, exercise), past medical history and family medical history. The variables were obtained through one-on-one surveys. Physical measurements were taken following the WHO standard protocol (23). These measurements included height, weight, waist circumference and blood pressure. The subjects were required to fast for at least 8 hours before blood samples were taken by a nurse (24). The blood samples were analyzed within 4 hours of collection. Automated biochemical analyzers were used to measure the levels of TC, TG, LDL-C, HDL-C, ALT and AST. TC and TG levels were estimated via enzymatic methods with commercially available reagents, whereas ALT, AST, HDL-C and LDL-C were measured via a timed endpoint colorimetric method. FBG levels were determined via glucose oxidase measurements (25).

### Exposure variables and outcomes

The exposure variable in this study was the AIP, which was defined as log10^[TG/HDL-C], and TG and HDL-C are expressed in mmol/L (13). The study subjects were divided into four groups based on the quartiles of AIP values at baseline (wave 0): group 1 (< -0.167), group 2 (-0.167 to < 0.012), group 3 (0.012 to < 0.194) and group 4 (≥0.195). The outcome event was a diagnosis of T2D during the follow-up period, and the time of the discovery of diabetes was recorded. According to other previous studies and the American Diabetes Association (7, 26), T2D can be diagnosed if one or more of the following criteria are met: (1) FBG ≥7.0 mmol/L, (2) random glucose ≥11.1 mmol/L, (3) glycated hemoglobin (HbA1c)≥6.5%, (4) self-reported diagnosis of diabetes by a physician, including the time of diagnosis, (5) receiving glucose-lowering therapy, including traditional Chinese medicine and modern Western medicine, or (6) receiving other treatments for diabetes or hyperglycemia.

### Potential confounding variables

Several variables previously identified as influencing factors for T2D were included as confounders in the study. These included baseline sociodemographic characteristics, lifestyle and behavior variables, physical measurements and biological indicators. Sociodemographic characteristics included sex, age, marital status (married or other), educational level (primary school and below, middle school, high school and above), family history of T2D, and history of hypertension. Hypertension history was self-reported as physician-diagnosed hypertension or the use of antihypertensive medication (27). Lifestyle and behaviors considered included smoking and alcohol consumption. The physical measurements included BMI, waist circumference, SBP, and DBP. BMI was calculated as weight (kg) divided by the square of height (m). Height and weight measurements were taken with participants removing shoes, hats, and wearing light clothing, with accuracies of 0.1 cm for height and 0.1 kg for weight. According to the WHO standard for BMI classification in Chinese populations, BMI was categorized into four groups: underweight (BMI <18.5 kg/m²), normal weight (BMI 18.5–23.9 kg/m²), overweight (BMI ≥24.0 kg/m²), and obese (BMI ≥28.0 kg/m²) (28). Waist circumference was measured at the midpoint of the midaxillary line between the lower edge of the costal arch and the midpoint of the iliac crest line via a 1.5 m tape (29). Biological indicators included primarily Hb, total cholesterol (TC), low-density lipoprotein cholesterol (LDL-C), aspartate transaminase (AST), and alanine aminotransferase (ALT).

### Participants and public involvement

Participants or the public were not included in the design, conduct, reporting, or dissemination plans of our research.

### Statistical analysis

AIP was categorized into four groups according to the quartile level of the subjects’ AIP values (group 1 was ≤25th percentile, group 2 ranged from >25th to 50th percentile, group 3 ranged from >50th to 75th percentile, and group 4 was >75th percentile). Continuous variables that followed a normal distribution are described as the means ± standard deviations, and one-way *ANOVA* was used for comparisons. The *post hoc Bonferroni* test was utilized for subgroup comparisons after ANOVA. Continuous variables that did not follow a normal distribution are described as medians (interquartile ranges *(IQRs*)) and *were compared with the Kruskal–Wallis* test. Frequencies (percentages) are presented for categorical variables and were compared with the *chi-square* test. Confounders known to be traditional or suspected risk factors for T2D.

For the longitudinal data analysis, we calculated the incidence of T2D per 1,000 person-years on the basis of the T2D diagnosis. The *Kaplan–Meier* method was used to compare the cumulative incidence. We utilized the *Cox* proportional hazards model to calculate *the hazard ratio (HR)* and *95% confidence interval (CI)* to assess the association between the AIP and the incidence of new-onset T2D. Model 1 was unadjusted, whereas Model 2 was adjusted for age, sex, education level, marital status, smoking status, alcohol consumption, BMI, and waist circumference. Model 3 included additional variables such as Hb, hypertension, SBP, DBP, FBG, TC, AST, ALT and LDL-C based on Model 2. In addition, we explored and compared the diagnostic values of the AIP, TG and HDL-C for the diagnosis of new-onset T2D by receiver operating characteristic (ROC) curve analysis via the area under the curve (AUC) and *Youden’s index*. Furthermore, the effect–dose–response relationship between the AIP and the risk of new-onset T2D was evaluated via a GAM based on a restricted cubic spline approach. Subgroup analyses were conducted via the *Cox* proportional hazards model with full adjustment for each subgroup. The associations between AIP and new-onset T2D were assessed across different age groups (65–69 years and ≥70 years), genders, educational levels (primary school and below, middle school, high school and above), smoking status (never, yes, quit), and alcohol consumption status (no, yes). Finally, participants who died during the follow-up period were excluded from the sensitivity analysis.

All the statistical analyses in the study were conducted via IBM SPSS (Version 26.0) and R version 4.4.1 (R Foundation for Statistical Computing). P< 0.05 was considered statistically significant in this study.

## Results

### Baseline characteristics of 18295 participants by AIP group

The study ultimately included 18,295 participants, of which 10,233 were females (55.93%) and 8,062 were males (44.07%). The mean age was 71.04 ± 4.86 years (ranging from 65 to 99 years). A total of 54.71% of the participants had a primary education or below. In terms of lifestyle factors, 1,815 participants (9.92%) were current smokers, and 1,889 (10.33%) were current alcohol consumers. Notably, the prevalence of hypertension among the population was 52.10%.

We categorized the AIP values into four groups on the basis of the quartiles of the AIP values. **Table 1** presents the sociodemographic and clinical characteristics of participants with different AIP levels at baseline. Most variables were significantly different between the groups. Compared with those with low AIP levels, a greater proportion of subjects with high AIP levels were female, had higher education levels, had hypertension and had a family history of T2D. Additionally, subjects with high AIP levels had a higher BMI; waist circumference; and SBP, DBP, Hb, ALT, AST, TC, TG, HDL-C and LDL-C levels (**Table 1**). The results of the *post hoc Bonferroni* test are shown in **Supplementary Table S1**.

**Table 1:** Demographic and clinical characteristics of participants by baseline AIP group.

Characteristics	Group1 (<-0.167)	Group2 (-0.167 to < 0.012)	Group3 (0.012 to < 0.194)	Group4 (≥ 0.195)	*P value*
Participants	4574	4575	4573	4573	
Age ^a^, years	71.22 ± 5.05	71.14 ± 4.93	70.96 ± 4.81	70.83 ± 4.62	0.001
BMI ^a^, kg/m2	22.63 ± 3.12	23.92 ± 3.01	24.48 ± 2.98	24.96 ± 2.96	<0.001
Waist circumference ^a^, cm	82.41 ± 8.87	86.17 ± 8.43	87.75 ± 8.19	89.20 ± 8.08	<0.001
Female ^c^, *n* (%)	2488 (54.39)	2511 (54.89)	2614 (57.16)	2620 (57.29)	0.005
Married ^c^, *n* (%)	4426 (96.76)	4442 (97.09)	4454 (97.40)	4447 (97.24)	0.308
Educational level ^c^, *n* (%)					
Primary school and below	2591 (56.65)	2533 (55.37)	2450 (53.58)	2436 (53.27)	0.003
Middle school	1362 (29.78)	1346 (29.42)	1412 (30.88)	1471 (32.17)	
High School or above	621 (13.57)	696 (15.21)	711 (15.54)	666 (14.56)	
Smoking ^c^					
No	3697 (80.83)	3598 (78.64)	3675 (80.36)	3615 (79.05)	0.153
Yes	429 (9.38)	475 (10.38)	445 (9.73)	466 (10.19)	
Quit	448 (9.79)	502 (10.97)	453 (9.91)	492 (10.76)	
Alcohol consumption ^c^					
No	4076 (89.11)	4083 (89.25)	4122 (90.14)	4125 (90.20)	0.179
Yes	498 (10.89)	492 (10.75)	451 (9.86)	448 (9.80)	
With diabetic family history ^c^, n (%)	106 (2.32)	135 (2.95)	174 (3.80)	160 (3.50)	<0.001
With hypertension ^c^, n (%)	2007 (43.88)	2334 (51.02)	2536 (55.46)	2654 (58.04)	<0.001
Clinical characteristics					
SBP ^a^, mmHg	132.04 ± 17.63	133.52 ± 16.90	134.82 ± 16.90	135.56 ± 16.34	<0.001
DBP ^a^, mmHg	77.34 ± 10.18	78.35 ± 9.92	78.84 ± 9.85	79.50 ± 9.62	<0.001
Hb ^a^, g/L	133.98 ± 16.36	136.41 ± 18.47	137.40 ± 15.93	138.33 ± 16.40	<0.001
FBG ^a^, mmol/L	5.33 ± 1.02	5.47 ± 1.14	5.64 ± 1.27	5.83 ± 1.47	<0.001
ALT ^b^, u/L	17.00 (13.00,22.90)	18.00 (14.00,24.16)	19.00 (14.40,26.00)	21.00 (15.90,29.60)	<0.001
AST ^b^, u/L	23.50 (20.00,28.00)	23.00 (20.00,28.30)	23.10 (20.00,29.00)	24.00 (20.00,30.00)	0.014
TC ^a^, mmol/L	5.01 ± 1.14	5.09 ± 1.29	5.15 ± 1.18	5.17 ± 1.11	<0.001
TG ^a^, mmol/L	0.83 ± 0.23	1.18 ± 0.26	1.58 ± 0.33	2.81 ± 0.59	<0.001
HDL-C ^a^, mmol/L	1.70 ± 0.49	1.39 ± 0.28	1.25 ± 0.24	1.10 ± 0.22	<0.001
LDL-C ^a^, mmol/L	2.78 ± 0.87	2.96 ± 0.95	3.04 ± 0.95	2.96 ± 0.98	<0.001

^a^ANOVA test; ^b^Kruskal–Wallis test; ^c^chi-square test.

### The incidence of new-onset T2D

**Table 2** shows the incidence of new-onset T2D among subjects during the follow-up period from 2021–2022. The overall incidence rate of new-onset T2D was 7.11% (95% *CI*: 6.74%, 7.48%). The cumulative incidence rates of new-onset T2D for groups 1 to 4 were 4.15% (95% *CI*: 3.57%, 4.73%), 6.12% (95% *CI*: 5.43%, 6.81%), 7.70% (95% *CI*: 6.93%, 8.47%), and 10.45% (95% *CI*: 9.56%, 11.34%), respectively. Furthermore, the incidence of new-onset T2D among subjects was 35.39/1000 person-years. The incidence rates for Groups 1 to 4 were 20.86, 30.70, 38.70, and 52.38 per 1,000 person-years, respectively. Notably, the incidence rate of new-onset T2D increased with increasing AIP, demonstrating a significant trend (*P-trend* < 0.001).

**Table 2:** The incidence rate of new-oneset of T2D over the duration of follow-up.

AIP	Participants (n)	T2D events (n)	Cumulative incidence (95% *CI*) (%)	Per 1,000 person-year
Total	18295	1300	7.11 (6.74,7.48)	35.39
AIP Group				
Group 1 (<-0.167)	4574	190	4.15 (3.57,4.73)	20.86
Group 2 (-0.167 to < 0.012)	4575	280	6.12 (5.43,6.81)	30.70
Group 3 (0.012 to < 0.194)	4573	352	7.70 (6.93,8.47)	38.70
Group 4 (≥ 0.195)	4573	478	10.45 (9.56,11.34)	52.38
*P-trend*			<0.001	<0.001

### Risk of new-onset T2D and AIP group

**Table 3** presents the results of Cox proportional hazards regression models, which include *the hazard ratio (HR)* and *95% confidence interval (CI)*, to examine the associations between AIP groups and the incidence of new-onset T2D. The risk of new-onset T2D significantly increases with increasing AIP, regardless of the inclusion of covariates. Furthermore, when AIP was divided into four groups based on quartiles, the risk of new-onset T2D was positively increased in the higher AIP groups (from Group 2 to Group 4) compared with the lowest AIP group (Group 1) (P trend < 0.001). In Model 3, the risk of new-onset T2D in Groups 2–4 was 26% (95% *CI*: 4%, 52%), 40% (95% *CI*: 17%, 68%), and 57% (95% *CI*: 31%, 87%) greater than that in Group 1, respectively. **Table 3** indicates a strong positive correlation between AIP scores and the risk of new-onset T2D.

**Table 3:** Longitudinal association between new one-set T2D and AIP group.

AIP Group	*HRs* (95% *CI*) of T2D					
	Model1	*P value*	Model2	*P value*	Model3	*P value*
Group 1 (< -0.167)	*reference*		*reference*		*reference*	
Group 2 (-0.167 to < 0.012)	1.49 (1.24,1.79)	<0.001	1.40 (1.17, 1.69)	<0.001	1.26 (1.04,1.52)	0.001
Group 3 (0.012 to < 0.194)	1.89 (1.58,2.25)	<0.001	1.74 (1.45,2.08)	<0.001	1.40 (1.17,1.68)	<0.001
Group 4 (≥ 0.195)	2.61 (2.20,3.08)	<0.001	2.36 (1.99,2.80)	<0.001	1.57 (1.31,1.87)	<0.001
*P-trend*	<0.001		<0.001		<0.001	

Model 1, no confounders were included; model 2, age, gender, educational level, marital status, smoking, alcohol consumption, BMI, and waist circumference were included. Model 3, Hb, hypertension, SBP, DBP, FBG, TC, AST, ALT and LDL-C based on Model 2.

**Figure 2:**
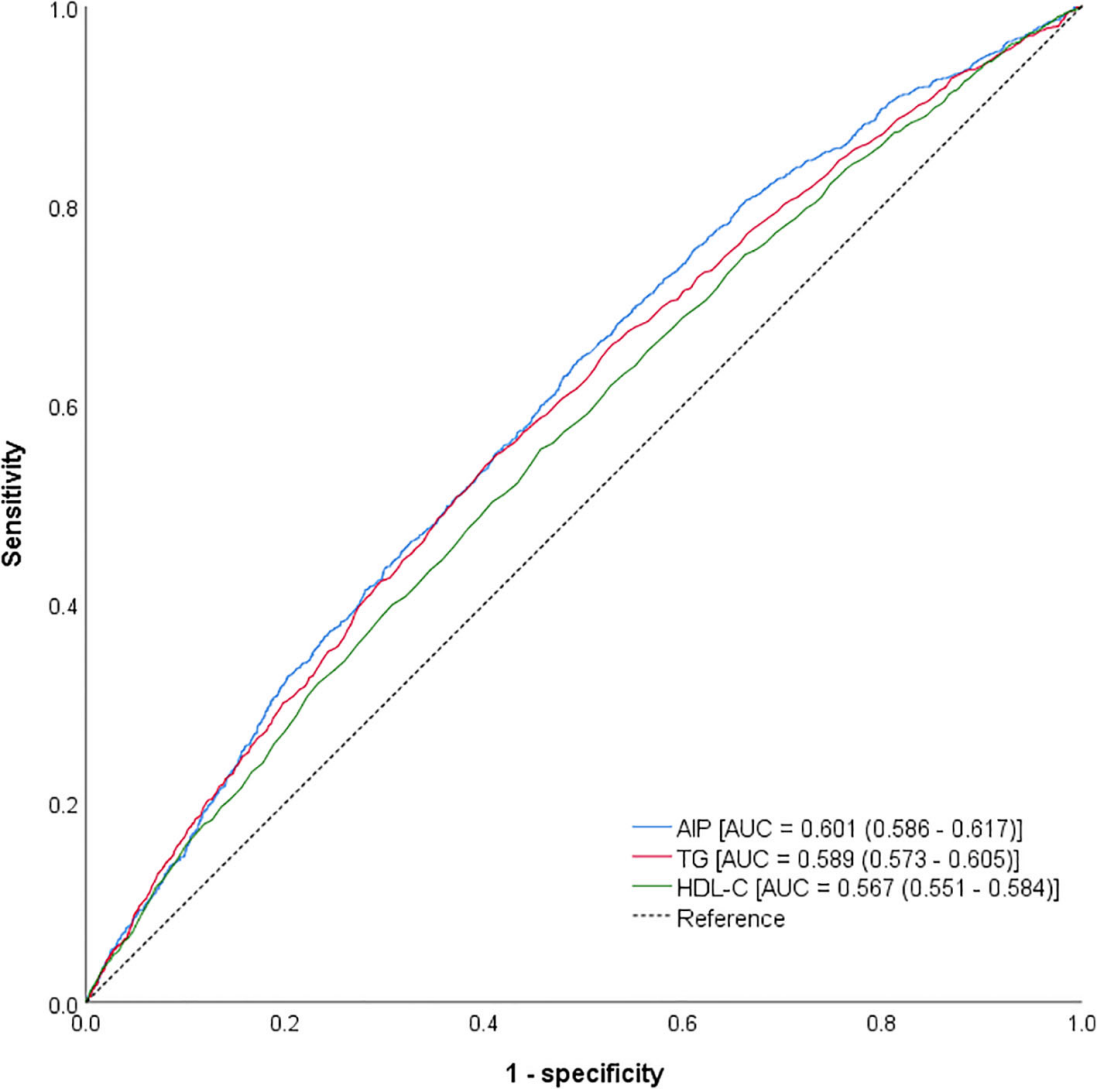
The ROC curves for AIP, TG, and HDL-C values for diagnosed new-onset T2D.

The *ROC* curves of the AIP, TG, and HDL-C values are presented in **Figure 2**. The AUCs of the AIP, TG, and HDL-C values were 0.601 (0.586–0.617; p<0.001), 0.589 (0.573–0.605; p<0.001), and 0.567 (0.551–0.584; P<0.001), respectively. Furthermore, we calculated the *Youden index* to determine the cutoff values. The best cutoff values for the AIP, TG and HDL-C were 0.355, 1.495 and 1.235, respectively. The corresponding sensitivities were 46.69%, 46.08%, and 49.77%, and the corresponding specificities were 89.45%, 40.14%, and 40.76%, respectively.

### Dose-response relationship between AIP and risk of new-onset T2D

**Figure 3:**
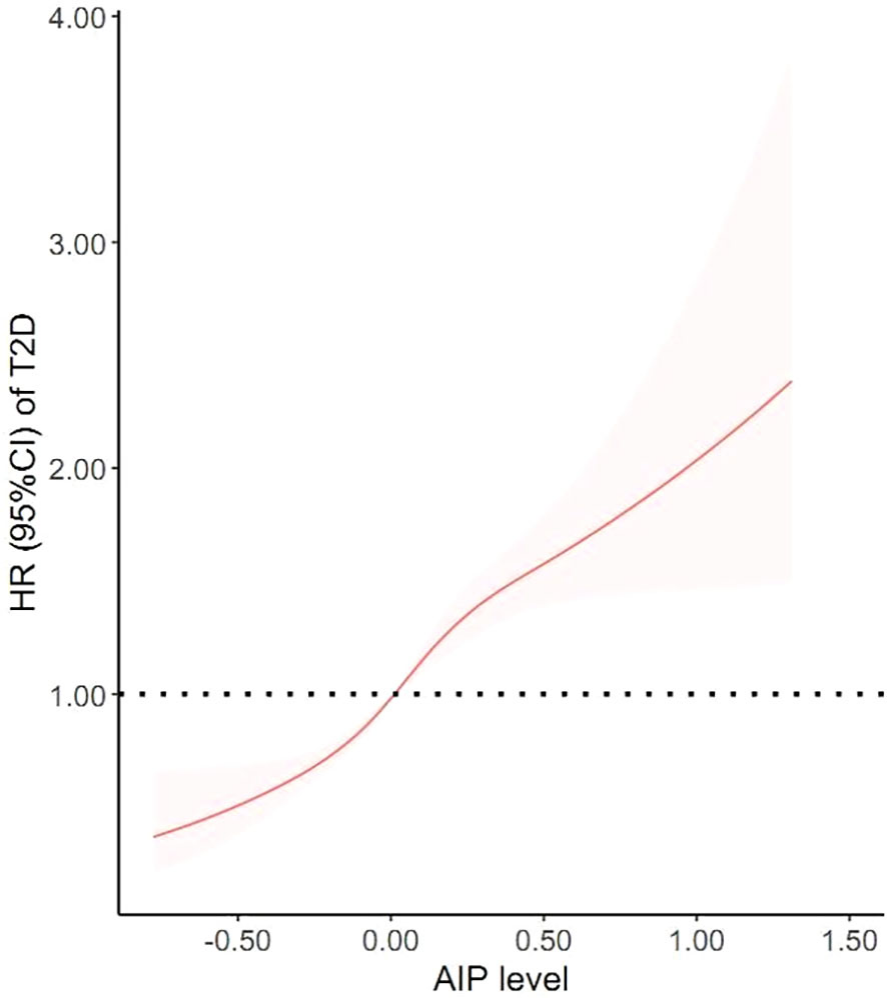
The nonlinear relationship between AIP and incidence of new-onset T2D.

After adjusting for confounders, a nonlinear association between the AIP and the risk of new-onset T2D was examined via a GAM based on a restricted cubic spline (*P-*nonlinear=0.031) (**Figure 3**). A threshold effect analysis revealed that the risk of new-onset T2D increased by 2.98 times for each one-unit increase in the AIP when the AIP was ≤0. Conversely, the risk of new-onset T2D increased by 1.95 times for each one-unit increase in the AIP when the AIP was > 0 (*P* < 0.05). **Table 4** presents the results of the two-piecewise Cox proportional hazards regression model.

**Table 4:** The result of two-piecewise Cox proportional hazards regression model.

AIP	*HR(95%CI)*	*P value*
Fitting model by standard Cox proportional hazards regression	2.50(2.05,3.04)	<0.001
Fitting model by two-piecewise Cox proportional hazards regression		
Infection points of AIP	0.00	
≤0.00	2.98(1.47,6.01)	0.002
>0.00	1.95(1.40,2.71)	<0.001

### Subgroup analysis and sensitivity analysis

In the subgroup analysis stratified by age (65–69 years and ≥70 years), sex (female and male), educational level (primary school and below, middle school, high school and above), the relationships between the AIP group and the risk of new-onset T2D still existed in most subgroups after other confounding factors were included (**Table 5**). To verify the robustness of the findings, we excluded 150 subjects who died during the follow-up period and repeated all analyses, and the relationship between the AIP and new-onset T2D still existed.

**Table 5:** Subgroup analysis of hazard ratios (95% *CI*) of depressive symptoms trajectories for T2D.

Subgroup	Group1 (<-0.167)	Group2 (-0.167 to <0.012)	Group3 (0.012 to <-0.194)	Group4 (≥0.195)	*P value*
Age, years					
65-69	*reference*	1.34 (1.03, 1.76)	1.42 (1.10, 1.85)	1.60 (1.25, 20.6)	<0.001
≥70	*reference*	1.19 (0.92, 1.82)	1.42 (1.10, 1.82)	1.60 (1.26, 2.04)	<0.001
Sex					
Male	*reference*	1.31 (1.01, 1.72)	1.50 (1.14, 1.97)	1.82 (1.40, 2.37)	<0.001
Female	*reference*	1.50 (1.16, 1.94)	1.45 (1.13, 1.85)	2.00 (1.58, 2.52)	<0.001
Educational level					
Primary school and below	*reference*	1.47 (1.15, 1.89)	1.71 (1.34, 2.17)	1.79 (1.41, 2.26)	<0.001
Middle school	*reference*	0.82 0.96, 1.36)	1.04 (0.74, 1.47)	1.43 (1.04, 1.97)	0.011
High school or above	*reference*	1.18 (0.71, 1.94)	1.45 (0.90, 2.35)	1.36 (0.84, 2.22)	0.433

Adjustment factors included age, gender, educational level, marital status, smoking, alcohol consumption, BMI, waist circumference, hypertension, SBP, DBP, Hb, FBG, ALT, AST, TC, and LDL-C.

## Discussion

This study is the first to analyze the association between AIP levels and the risk of new-onset T2D in the elderly population via a large longitudinal survey. A strong positive association between the AIP and new-onset T2D was still valid after the participants were divided into four groups on the basis of the quartiles of the AIP level. Furthermore, the results of the GAM analyses revealed a nonlinear dose–response relationship between the baseline AIP and the risk of new-onset T2D, demonstrating that the risk of new-onset T2D increased with increasing baseline AIP. These findings suggest that the AIP could serve as a valuable indicator for monitoring and preventing new-onset T2D.

AIP was defined as log10^[TG/HDL-C] by Dobiásová and Frohlich in 2001 (8). It was initially introduced as a novel and straightforward biomarker for predicting CVD. Recent studies have confirmed that the AIP is positively associated with various metabolic diseases, including obesity (30), prediabetes (18), T2D (19), and metabolic syndrome (31). We found a significant positive relationship between the AIP and several parameters, such as BMI, waist circumference, SBP, DBP, Hb, FBG, ALT, AST, TC and LDL-C. Furthermore, individuals with hypertension or a family history of T2D presented higher AIP levels, which aligns with the results of other studies (17–19, 30). Interestingly, we also observed that individuals with higher education levels and women had elevated AIP levels, which is not entirely consistent with previous research (17, 32, 33). This discrepancy may be attributed to differences in subject characteristics. Only 10.72% of the subjects had a high school education or above, while the subjects in other studies had higher levels of education.

An increasing number of studies have focused on the association between the AIP and T2D. However, most studies are cross-sectional surveys with limited sample sizes (18–20). The study evaluated the relationship between AIP levels and new-onset T2D using data from a large longitudinal survey and has strong credibility. These findings indicate that the risk of new-onset T2D positively increases with increasing AIP. After adjusting for confounding variables, when the subjects were divided into four groups on the basis of quartiles of AIP levels, the risk of new-onset T2D was 1.26, 1.40 and 1.57 times greater for subjects in the second, third, and fourth groups, respectively, than for those in the first group. We also found a positive association between the AIP and the risk of new-onset T2D via GAM analyses, which was consistent with previous cross-sectional findings (18, 19). A meta-analysis demonstrated that the AIP is a more reliable predictor of T2D than other lipid components are (34). Additionally, a cohort study conducted in a middle-aged and elderly Chinese population evaluated the associations between longitudinal AIP conversion patterns over time and new-onset T2D. During the follow-up period, individuals who maintained high AIP levels had a greater risk of new-onset T2D than did those who maintained a low AIP pattern (16). These studies suggest that early intervention in lipid management (such as raising awareness of dyslipidemia, promoting a healthy diet, and encouraging regular exercise) should be prioritized to prevent new-onset T2D. In the subgroup analyses, we identified several findings that differed from those of previous studies. A cross-sectional study of adults in the United States demonstrated a positive association between AIP and T2D among all age groups, with significant differences observed between sexes (18). Conversely, a cohort study in Taiwan Province, China, revealed that the AIP was positively associated with T2D only in the 40–64 year age group, and no sex differences were reported (15). Our study focused on elderly individuals, and we did not find age or sex differences in the associations between AIP and new-onset T2D. These discrepancies may be attributed to the distinct characteristics of the study populations. Additionally, we found that the AIP exhibited inconsistent associations with the risk of new-onset T2D among subgroups differentiated by literacy level. These inconsistencies may be related to the lower literacy levels of our study population, and the findings underscore the necessity for further longitudinal studies to validate the results.

The mechanisms underlying the development of AIP and diabetes remain unclear. It is currently hypothesized that their association can be explained by a ‘vicious circle’, i.e., dyslipidemia-insulin resistance-hyperinsulinemia (35). High levels of TG contribute to diabetes by competing with glucose for cellular entry, thereby reducing the number and activity of insulin receptors on adipocytes and preventing insulin from binding to these receptors (35). Additionally, reduced levels of HDL-C can impair pancreatic β-cell function, leading to decreased insulin secretion and sensitivity (36). Abnormal lipid levels can also induce inflammation, endoplasmic reticulum stress, and lipotoxicity, all of which contribute to insulin resistance (IR) and subsequently to the development of diabetes (37). Therefore, early interventions aimed at disrupting the vicious cycle may be effective in preventing new-onset diabetes.

Previous studies evaluating the association between AIP and T2D were primarily cross-sectional studies, including adult populations with small sample sizes (fewer than 400 participants). We utilized longitudinal survey data with a larger sample for the first time to explore the longitudinal association between the AIP and new-onset T2D in older adults. The approach facilitated more robust and representative conclusions. Moreover, the definition of T2D events in BaHLS was comprehensive, incorporating not only self-reported medical histories but also biological indicators such as fasting glucose, random glucose, and glycated hemoglobin. This methodology effectively minimized the underdiagnosis of diabetes. Additionally. All the surveys were conducted face-to-face by trained professionals via structured questionnaires. Physical examination and laboratory test indicators were collected and verified by medical personnel following standardized protocols to ensure data consistency and reliability. Finally, both subgroup analyses and sensitivity analyses were performed, further confirming the robustness of the positive association between AIP and T2D.

Despite the strengths of this study, several limitations should be considered. First, although we adjusted for a set of potential confounders on the basis of prior knowledge, some additional confounders, such as physical activity, dietary intake and genetic susceptibility, were not considered in our research. Second, some characteristics were based on questionnaire surveys, inevitably leading to recall bias. Moreover, although the correlation between the AIP and T2D in the longitudinal study was stronger than that in the cross-sectional study, we could not interpret the potential biological mechanisms involved. Therefore, further experimental studies are necessary to confirm this association. Third, although the study suggests that the AIP is a more reliable predictor of T2D than other lipid components are, its predictive performance is not highly efficient. Further studies need to effectively combine the AIP with other indicators, such as HbA1c, waist circumference, and BMI, to enhance the predictive efficacy for new-onset T2D.

## Conclusion

This longitudinal study revealed a positive correlation between the AIP and new-onset T2D among elderly individuals, providing new evidence for a causal relationship. In the context of China’s rapidly aging population, increasing physical activity and nutrition interventions are conducive to improving lipid levels, which further contributes to decreasing and delaying new-onset T2D.

## Data Availability

The original contributions presented in the study are included in the article/**Supplementary Material**. Further inquiries can be directed to the corresponding author.

## Supplementary material

Supplementary Table 1
*Post hoc Bonferroni* test of demographic and clinical characteristics of participants by baseline AIP group. ^a^compare with Group 1, *P* < 0.05; ^b^compare with Group 2, *P* < 0.05; ^c^compare with Group 3, *P* < 0.05; ^d^compare with Group 4, *P* < 0.05.
