# Supplementary material for: Association between atherogenic index of plasma and new onset of type 2 diabetes among elderly in China: a longitudinal study
Source: Front Endocrinol (Lausanne). 2025 Oct 15;16:1632400. doi: 10.3389/fendo.2025.1632400 (PMC12568405; doi:10.3389/fendo.2025.1632400)
Supplement: Supplementary Table 1 — Post hoc Bonferroni test of demographic and clinical characteristics of participants by baseline AIP group. acompare with Group 1, P < 0.05; bcompare with Group 2, P < 0.05; ccompare with Group 3, P < 0.05; dcompare with Group 4, P < 0.05. [file Table1.docx]

**Table S1.** *Post hoc Bonferroni* test of demographic and clinical characteristics of participants by baseline AIP group

| Characteristics | Group1 (<-0.167) | Group2  (-0.167 to < 0.012) | Group3  (0.012 to < 0.194) | Group4 (≥ 0.195) |
| --- | --- | --- | --- | --- |
| Age, years | 71.22±5.05^cd^ | 71.14±4.93^cd^ | 70.96±4.81^ab^ | 70.83±4.62^ab^ |
| BMI, kg/m2 | 22.63±3.12^bcd^ | 23.92±3.01^acd^ | 24.48±2.98^abd^ | 24.96±2.96^abc^ |
| Waist circumference, cm | 82.41±8.87^abc^ | 86.17±8.43^ad^ | 87.75±8.19^ad^ | 89.20±8.08^abc^ |
| Clinical characteristics |  |  |  |  |
| SBP, mmHg | 132.04±17.63^bcd^ | 133.52±16.90^ad^ | 134.82±16.90^ad^ | 135.56±16.34^abc^ |
| DBP, mmHg | 77.34±10.18^bcd^ | 78.35±9.92^ab^ | 78.84±9.85^ab^ | 79.50±9.62^abc^ |
| Hb, g/L | 133.98±16.36^bcd^ | 136.41±18.47^ad^ | 137.40±15.93^ad^ | 138.33±16.40^abc^ |
| FBG, mmol/L | 5.33±1.02^bcd^ | 5.47±1.14^acd^ | 5.64±1.27^abd^ | 5.83±1.47^abc^ |
| ALT, u/L | 17.00(13.00,22.90)^bcd^ | 18.00(14.00,24.16)^acd^ | 19.00(14.40,26.00)^abd^ | 21.00(15.90,29.60)^abc^ |
| AST, u/L | 23.50(20.00,28.00)^d^ | 23.00(20.00,28.30)^d^ | 23.10(20.00,29.00) | 24.00(20.00,30.00)^ab^ |
| TC, mmol/L | 5.01±1.14^cd^ | 5.09±1.29 | 5.15±1.18^a^ | 5.17±1.11^a^ |
| TG, mmol/L | 0.83±0.23^bcd^ | 1.18±0.26^acd^ | 1.58±0.33^abd^ | 2.81±0.59^abc^ |
| HDL-C, mmol/L | 1.70±0.49^bcd^ | 1.39±0.28^acd^ | 1.25±0.24^abd^ | 1.10±0.22^abc^ |
| LDL-C, mmol/L | 2.78±0.87^bcd^ | 2.96±0.95^ac^ | 3.04±0.95^abd^ | 2.96±0.98^ac^ |

^a^ compare with Group 1, *P*<0.05; ^b^ compare with Group 2, *P*<0.05; ^c^ compare with Group 3, *P*<0.05; ^d^ compare with Group 4, *P*<0.05.
